# Automated spectral decomposition and reconstruction of optical properties using a mixed autoencoder approach

**DOI:** 10.1117/1.JBO.30.4.047001

**Published:** 2025-04-09

**Authors:** Dongqin Ni, Marine Amouroux, Walter Blondel, Martin Hohmann

**Affiliations:** a Friedrich-Alexander-Universität Erlangen-Nürnberg, Institute of Photonic Technologies, Erlangen, Germany; b Erlangen Graduate School in Advanced Optical Technologies, Erlangen, Germany; c Université de Lorraine, CNRS, CRAN UMR 7039, Vandoeuvre-Lès-Nancy, France

**Keywords:** machine learning, optical properties, autoencoder, explainable neural networks

## Abstract

**Significance:**

Investigating optical properties (OPs) is crucial in the field of biophotonics, as it has a broad impact on understanding light-tissue interactions. However, current techniques, such as inverse Monte Carlo simulations (IMCS), have limitations in extracting detailed information about the spectral behavior of microscopic absorbers and scatterers.

**Aim:**

We aim to develop a customized autoencoder neural network (ANN) that can automatically identify the spectral behavior of each microscopic absorber and scatterer responsible for generating OP.

**Approach:**

The ANN is designed to compute OP from measurements, in which the bottleneck corresponds to the number of absorbers and scatterers. The presented ANN functions asymmetrically and computes the OP using a linear combination of absorbers and scatterers. Validation was conducted using intralipid as a scatterer and ink as an absorber.

**Results:**

The employment of the decoder weights facilitated the successful extraction of the spectral shape of every constituent, demonstrating the effectiveness of the ANN in extracting detailed information about the spectral behavior of absorbers and scatterers. At the same time, the OP can be predicted with high precision.

**Conclusions:**

The presented ANN is a viable tool for extracting the spectral behavior of absorbers and scatterers without the need for prior knowledge of these components in the test and training data. Potential future applications could include the extraction of relative concentrations of constituents in tissue.

## 1 Introduction

For modeling the connection among the total transmission (TT), the total reflection (TR), and the optical properties (OP), various techniques have been available for several decades,^1^ with integrating spheres in combination with inverse Monte Carlo simulations (IMCS) being the most advanced for *ex vivo* contexts.^2^^,^^3^ Thereby, IMCS provided an advantage in accuracy over older techniques such as Kubelka-Munk (KM)^4^ or inverse adding doubling (IAD).^5^^,^^6^ An alternative is the usage of neural networks (NN)^7^^–^^11^ which has become more commonly used recently.^12^^–^^15^ However, the application of NNs has the same inherent limitation as the previously discussed methods, namely that the spectral behavior and concentration of constituents such as ink, hemoglobin, or melanin for absorbers and intralipid, titanium oxide, collagen, or cell organelles for scatterers, which are the origin of the OP cannot be accessed. Nevertheless, Ni et al.^16^ demonstrated that one possible benefit of NN lies in its capacity to yield precise results for optical configurations that were previously deemed intractable using conventional theory. The question of how intrinsic information of the constituents can be derived remains unanswered. This issue has been partially addressed by Ni et al.^17^ through the use of customized autoencoder neural networks (ANNs), although some systematic errors were observed.

An ANN typically consists of an encoder network that compresses the data into a few neurons and a decoder that subsequently recovers these data. The compressed data are referred to as the latent space, which is typically high-dimensional, complex, and nonlinear.^18^ However, under the right conditions, the latent space can become interpretable, as demonstrated in the case of anterior segment optical coherence tomography images^19^ with the aid of variational ANN.^20^ Ni et al.^17^ demonstrated that a mixed ANN comprising a deep encoder with numerous hidden layers and a linear decoder enables the problem at hand to be linearised, aligning the encoding neurons and the weights of the decoder with the relative concentration and spectral response of the constituents of the sample, without the need for their prior knowledge in the training data. The term relative concentration is used as the concentrations from the ANN have to be calibrated once for the ANN after training. The proposed design is a modification of the design from Palsson et al.^21^ in which it was suggested as a new method for spectral unmixing of hyperspectral images.^22^

This study builds upon the findings of the previous investigation^17^ utilizing the ANN to address the identified shortcomings in the earlier study. Although it is known that by training an NN with measurements of TT and TR from a standard spectrophotometer as input and the absorption coefficient ($\mu_{a}(\lambda )$) as well as the reduced scattering coefficient ($\mu_{s}^{\prime }(\lambda )$) as output, it can precisely predict the absorption and scattering coefficient.^15^^,^^16^ However, the proposed ANN system can derive precise and accurate predictions of the spectral response and relative concentration of each constituent without knowing it prior to the training of the ANN. In addition, the potential for generalization of the proposed system is demonstrated by adapting it to a more complex problem in which the scattering coefficient ($\mu_{s}$) and the g-factor are reconstructed by adding a second measurement of the same sample with a different thickness.

## 2 Methods

### 2.1 Experimental Parameters

The experimental parameters were the same as in our previous publication, and the measurement procedure is described in detail^16^ from which part of the experimental results were also used in the previous proceeding.^17^ For convenience a small summary is provided:

“The data is generated by measuring the total transmission (TT) and total reflection (TR) using a commercially available spectrophotometer (Shimadzu UV-3600 UV-VIS-NIR Spectrophotometer, Double Beam, Three Detector System, Japan) equipped with the LISR-3100 Integrating Sphere (IS) attachment.”^16^

The measurements were taken from two cuvettes with an internal thickness of either 5 mm or 10 mm in the spectral range of 500 to 800 nm with a step size of 2 nm.

“The sample comprises liquid phantoms made of intra-lipid (IL) from Fresenius Kabi and either Modena Red ink from MONTBLANC or Indian Ink from Winsor & Newton. A total of 496 samples were prepared with 16 different concentrations of IL, 16 different concentrations of red ink and 15 different concentrations of black ink. The OP of the IL were obtained from Aernouts et al.’s study.^23^ The ink’s characteristics were determined by previous measurements without any scatterers”^16^ which is a modification of the prior publication.^24^ From the measured data, all 496 measured spectra were used. Prior to the experiment, the extinction coefficient of the ink was calculated in samples without scatterers. These values serve as the ink reference in this study. The concentrations for IL range from 0.0125% to 4%. For the red and black ink, they range from 0.01% to 0.5% and 0.01% to 0.25%, respectively. All concentrations are expressed as the volume of the undiluted substance relative to the total volume.

### 2.2 Machine Learning

Two sets of experiments were performed: In the initial experiment, the spectrally resolved TT and TR for the 5-mm-thick cuvette served as input, whereas the spectrally resolved absorption and reduced scattering coefficients were used as output. In a second experiment, TT and TR were used from both cuvettes, and in addition to the absorption and reduced scattering coefficient, the scattering coefficient and the g-factor were used as output.

The complete data analysis was done in Python, using tensorflow^25^ for the NN and the training was performed on an RTX 3090 (Nvidia, California, United States). The data from the measurements were randomly divided into training and test data. Here, 10% of the data was randomly used as test data. The 90/10 split was chosen based on common practices in machine learning for initial proof-of-concept studies where data availability might be a constraint. Moreover, rerunning the code with different randomly selected test data does not alter the results significantly. The spectrally resolved TT and TR were used as input, and the spectrally resolved absorption and reduced scattering coefficients were used as output. For a standard NN structure, TT and TR are directly transformed into the final OP. However, the aim of this study is for the NN to automatically incorporate the absorption and scattering properties of the constituent from where it derives the final OP.

**Fig. 1:**
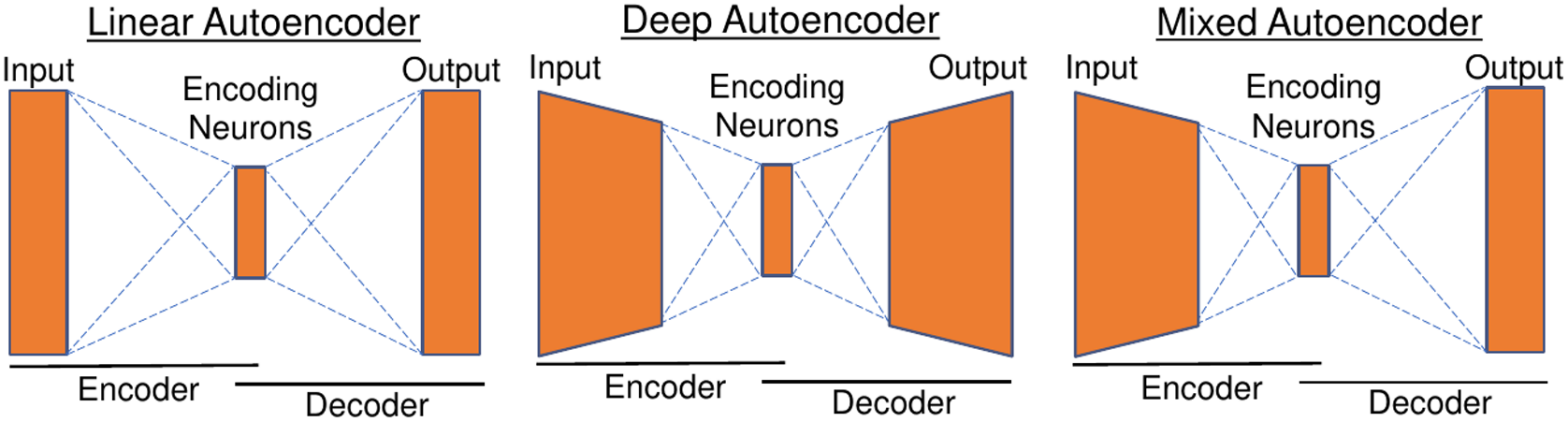
Basic principle of linear autoencoder (left), deep autoencoder (middle), and mixed autoencoder which is used in this study (right). A linear encoder or decoder is represented by a rectangle and a nonlinear encoder or decoder is represented by a trapezium.

The NN structure is identical in both experimental settings. In both cases, the TT and TR are directly transformed into the final OP. As previously mentioned, the objective is for the NN to automatically incorporate the absorption and scattering properties of the constituents into their structure to derive their final OP. For this reason, an ANN design is used as shown in Fig. 1 with n ($n\in N^{+}$) encoding neurons.

In general, the ANN approach can be divided into three cases: the linear ANN (Fig. 1 left) behaves similarly to the principle component analysis (PCA) with n first components. Although this allows an easy interpretation of the data, the constituent’s absorption and scattering properties cannot be derived in this way. The deep ANN (Fig. 1 center) is able to encode the OP. However, the neurons cannot be interpreted because the spectral response of a single encoding neuron changes depending on the value. In this study, both ANN designs are therefore combined: a nonlinear encoder with a linear decoder. This allows nonlinear problems to be transformed into linear relationships. It should be noted that, in contrast to the work of Palsson et al.,^21^ no autoencoding is performed in this study, as the input and output are not identical.

The overall structure of the neural network is outlined in Table 1. Initially, a convolutional layer is employed for data smoothing, followed by a flattening layer. After this step, the data passes through nine additional layers designed to optimize the prediction of the desired OP. The activation functions and kernel initializers are carefully selected to increase the likelihood of successful convergence. Although different architectures for the encoder are possible, the model’s performance is generally robust to these design choices.

**Table 1:** Layers and parameters of the used NN.

Layer number	Layer type	Number of neurons/other parameters	Activation	Kernel initializer
1	Convolution 1D	filters = 8, kernel size = 3	gelu	he_normal
2	Flatten	—	—	—
3	Dense	300	gelu	he_normal
4	Dense	200	tanh	LecunUniform
5	Dropout	rate = 0.04	—	LecunNormal
6	Dense	150	sigmoid	LecunUniform
7	Dropout	rate = 0.08	—	—
8	Dense	100	gelu	he_normal
9	Dense	70	tanh	—
10	Dense	3	gelu	LecunUniform
11	Dense	300/600, no bias, kernel weight restriction, kernel regularizer = L1L2	linear	ones

A key component of the neural network is the configuration of the final layer, which is critical for ensuring realistic convergence. In this layer, all initial weights are set to one to prevent the neurons describing the spectral response from prematurely converging to zero. In addition, the final layer has no bias, ensuring completely linear behavior. The completely linear design of the decoder ensures that twice the concentration of the sample would result in twice the concentration measured by the encoder. Hence, relative concentrations can be measured with the presented network. The network is also designed to separate the influence of scatterers and absorbers: photon weights are restricted such that a scatterer only affects scattering parameters ((reduced) scattering coefficient and g), whereas an absorber exclusively influences the absorption coefficient. This separation is achieved by constraining the decoding neurons’ kernels, setting the absorber’s weight to zero for scatterers, and vice versa. The number of encoding neurons in the tenth layer is set to three, corresponding to the number of constituents. The number of scatterers is set to one, and the number of absorbers is set to two, matching the constituents used in the experiments.

To further refine the network’s predictions, several loss functions are applied. First, L1L2 kernel regularization with parameters 0.02 and 0.05 penalizes the network for using multiple neurons to describe a single absorber, addressing a limitation from a previous study.^17^ Second, Pearson correlation loss is applied to the encoder output to ensure the accurate prediction of OP. An additional loss penalizes negative neuron weights, enforcing the physical constraint that concentrations represented by the encoder output must remain non-negative.

The optimization scheme is depicted in Table 2. The optimization was done in four steps with Adam as the optimizer. The overall strategy was to first train the NN to a local minimum and afterward leave this local minimum using a higher learning rate. Afterward, the NN is trained to slowly converge toward the desired minimum. The output of the encoding neurons is restricted to ensure physically realistic results. To achieve this, a second loss function is added to penalize negative values of the encoding neurons, as negative absorption or scattering is not possible.

**Table 2:** Optimizer strategy.

Optimizing step	Learning rate	$\beta_{1}$	$\beta_{2}$	ϵ
1	$1\times 10^{-5}$	0.8	0.9999	$1\times 10^{-7}$
2	$1\times 10^{-3}$	0.8	0.999	$1\times 10^{-10}$
3	$5\times 10^{-4}$	0.8	0.999	$1\times 10^{-10}$
4	$1\times 10^{-5}$	0.8	0.9999	$1\times 10^{-7}$

## 3 Results and Discussion

Each experiment yields two distinct outputs. First, it is essential to ascertain the degree of precision that can be achieved in the reconstruction of the OP. On the other hand, the precision of reconstruction of the constituent’s absorption and scattering properties needs to be discussed. For both experiments, the OP can be reconstructed with an $R^{2}$-value larger than 0.99 except for the g-factor. These results are in accordance with expectations. This discrepancy for the g-factor is attributed to the inability of the linear decoder to account for the different concentration-dependent variations in comparison to the concentration dependence of the scattering and reduced scattering coefficients. This issue can be resolved by enabling the bias in the decoder; however, this is not currently done as the bias would introduce unwanted nonlinearities in the decoder. Notwithstanding this limitation, the findings demonstrate that the mixed autoencoder represents an effective approach for the reconstruction of the OP. This result is consistent with previous studies that have demonstrated that the OP can be reconstructed with such precision using machine learning techniques.^15^^,^^17^

**Fig. 2:**
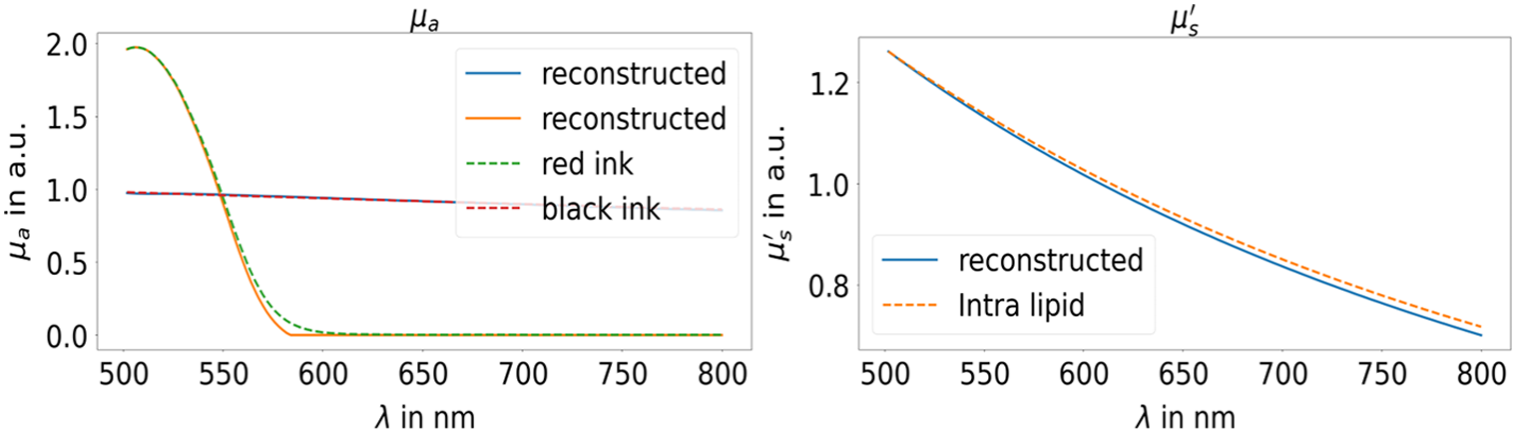
Reconstructed decoder weights representing the spectral response of the absorbers (left) and the scatterer represented by the reduced scattering coefficient (right) in comparison with the real data for the first experiment with a single cuvette.

Figure 2 depicts the reconstruction of the constituent’s absorption and scattering properties from the decoder weights in comparison to the real OP for the input transmission and reflectance of the 5-mm single cuvette. Overall, there is a high degree of agreement between the decoder weights and the OP. A minor discrepancy is observed for the red ink at around 570 nm, which can be attributed to the application of the L1L2 kernel regularisation.

**Fig. 3:**
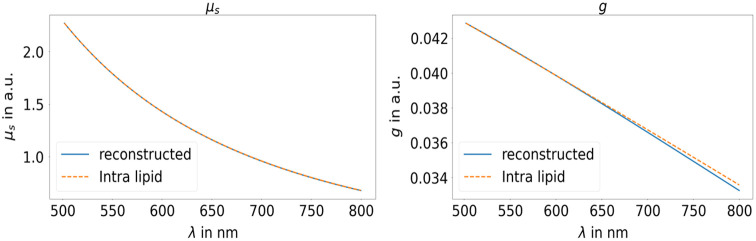
Reconstructed decoder weights representing the spectral response of the scatterer for the scattering coefficient (left) and the g-factor (right) in comparison with the real data for the second experiment with two cuvettes.

Figure 3 illustrates the reconstruction of the constituent’s scattering coefficient and the g-factor, based on the input of transmission and reflectance from both cuvettes. The results for the absorption and reduced scattering coefficients are identical to those in Fig. 2 and thus not shown. It can be seen that both properties can be derived with high precision despite the fact that the correct g-factor cannot be predicted by the mixed autoencoder design or in other words, the method can predict the shape of the OP, but not the absolute value.

Although the results are encouraging, it is crucial to consider the limitations of this study. It should be noted that only relative concentrations can be measured. This implies that the observed concentrations which are the output of the encoder are reconstructed in arbitrary units. Nevertheless, the completely linear design of the decoder, in conjunction with the excellent predictions of the OP, ensures that twice the concentration of the sample would result in twice the relative concentration measured by the encoder. However, the relative concentrations must be calibrated each time after the ANN is trained, as retraining does not allow it to converge to the same arbitrary concentrations. Moreover, the current ANN design assumes that each constituent can be classified exclusively as either an absorber or a scatterer. Although this assumption holds for red ink and intralipid, it is known that the black ink used exhibits some degree of scattering.^26^^,^^27^ This scattering effect may contribute to the observed discrepancies between the real values and the predicted reduced scattering coefficient in the first experiment, as well as the g-factor in the second experiment. Future work could address this issue by allowing the ANN to model materials with both scattering and absorbing properties while incorporating a regularization term that penalizes excessive use of such dual classifications.

Furthermore, the proof of concept was conducted with a single absorber at a time; consequently, the investigation of multi-absorber scenarios is imperative for future research. Despite this, it is anticipated that the model will function in this instance, as indicated by the findings from the field of remote sensing.^21^^,^^22^ Second, the NN can only accurately predict absorbers and scatterers that were present during the training process. However, prior knowledge of these constituents is not necessary.

## 4 Conclusion

This study demonstrates the effectiveness of the proposed ANN in extracting detailed information about the spectral behavior of microscopic absorbers and scatterers, which are critical to generating OP. The ANN design, combining a nonlinear encoder with a linear decoder, allows for automatic learning of intrinsic system properties without requiring prior knowledge of the constituent values. Unlike other state-of-the-art models, this approach provides direct interpretability of physical parameters. This is evident from the proportionality observed between the encoder weights and the spectral response, as well as the clear relationship between the encoding neuron values and the constituent relative concentrations. The ANN accurately reconstructs OP except g with an $R^{2}$-value exceeding 0.99 and is also capable of deriving the constituent’s absorption and scattering properties in scenarios where standard scattering theory would not predict such results. The decoder weights, representing the spectral responses of each constituent, are in close agreement with the real OP. Moreover, this work addresses and compensates for previously identified systematic errors, specifically the issue where the spectral contributions of the two absorbers could not be properly separated due to the network assigning similar weights to both. By implementing the L1L2 kernel regularization, we encouraged the network to use distinct neurons for each absorber, thus improving the separation and expanding the model’s generalizability to other complex problems.

A key contribution of this proof-of-concept study is demonstrating the ANN’s ability to accurately reconstruct the spectral properties of individual absorbers and scatterers in single-constituent systems with significantly reduced reliance on a priori knowledge compared with traditional methods. The proposed ANN design holds promise as a valuable tool in biophotonics, enabling precise extraction of spectral behavior in microscopic absorbers and scatterers, even in challenging *in vivo* or tissue-based settings where prior knowledge is unavailable. Future work should focus on extending the generalization capabilities by applying the ANN to tissue and developing methods for the automatic determination of the appropriate number of encoding neurons for varying systems. Furthermore, this ANN has the potential for broader applications beyond OP. For instance, it could be applied to imaging techniques to enable real-time mapping of physiological parameters such as blood oxygenation. In addition, in fluorescence imaging, the network could be adapted to retrieve fluorescent properties. Beyond biophotonics, the ANN could be utilized in fields such as atmospheric sensing, pollution tracking, or hyperspectral imaging to analyze material composition. Hence, it is expected that the proposed ANN can be generalized to fields beyond the reconstruction of OP and even biophotonics.

## Data Availability

The data are available upon reasonable request.
